# Synergistic and Antagonistic Effects of Hybridization and MWCNT Reinforcement on the Solid Particle Erosion of Glass/Carbon Fiber Composites

**DOI:** 10.3390/polym17182434

**Published:** 2025-09-09

**Authors:** Seyit Mehmet Demet

**Affiliations:** Mechanical Engineering Department, Engineering and Natural Science Faculty, Konya Technical University, Konya 42250, Türkiye; smdemet@ktun.edu.tr

**Keywords:** solid particle erosion, hybrid composites, MWCNTs

## Abstract

A systematic investigation into the solid particle erosion (SPE) of monolithic, sandwich-type hybrid and multi-scale (Multi Wallet Carbon Nanotube (MWCNT)-reinforced) glass/carbon fiber composites was performed confirming to the ASTM G76-18 standard, utilizing angular alumina erodent (~600 µm) at 34 m/s across key impingement angles of 30°, 45°, 60°, and 90°. The analysis reveals a profound performance dichotomy dictated by the governing wear mechanism. At the shear-dominated 30° angle, where maximum material loss was observed, hybridization consistently enhanced erosion resistance relative to both monolithic benchmarks. This synergy, however, contrasts sharply with the nuanced behavior under the 90° impact-dominant regime; here, although strategically hybridizing a brittle CFRP with tougher glass fibers reduced the erosion rate (ER) by a remarkable ~50%, this benefit was compromised by the matrix embrittlement induced by MWCNT incorporation. This work clarifies the difference between shear-dominated erosion in the ductile regime and fracture toughness under impact-dominated conditions.

## 1. Introduction

At the intersection of modern engineering design paradigms, driven by the dual imperatives of performance optimization and light-weighting [1,2], Polymer Matrix Composites (PMCs) have firmly established themselves as an indispensable class of materials [3]. Specifically, glass fiber-reinforced polymers (GFRPs) and carbon fiber-reinforced polymers (CFRPs) have spearheaded structural innovations across the aerospace, automotive, and wind energy sectors, owing to their exceptional specific strength and stiffness profiles [4,5]. The expanding spectrum of their applications, however, inevitably subjects these materials to demanding environmental and operational conditions.

Chief among these challenges is SPE, a surface degradation phenomenon characterized by the high-velocity impingement of solid particles (e.g., sand, dust, and other particulates) onto a material’s surface. In critical components—such as helicopter rotor blades, gas turbine compressor blades [6,7], and the leading edges of wind turbine blades [8]—erosion transcends a merely cosmetic concern, constituting a principal damage mechanism that jeopardizes structural integrity and curtails functional lifetime [9]. While the resultant material loss can degrade performance by altering aerodynamic profiles, a more critical consequence is that the surface and subsurface damage as a precursor to other failure modes. The micro-cracks and indentations generated by erosion serve as potent initiation sites for fatigue, potentially precipitating premature and catastrophic structural failure [10,11,12].

The erosion response of PMCs is governed by a complex interplay between their inherently anisotropic and heterogeneous microstructure and a suite of extrinsic parameters [13,14], most notably the kinetic energy and impingement angle of the erodent particles [15,16]. Consequently, erosion behavior is conventionally classified based on the impingement angle that elicits the peak ER. The response is thereby categorized as ductile when maximum material loss occurs at low angles (15–30°), semi-ductile at intermediate angles (30–60°), and brittle at normal incidence (~90°) [17,18,19].

Under low-angle impingement (15–30°), the erosion of ductile materials primarily occurs through mechanisms related to plastic deformation of the matrix, such as micro-cutting and ploughing [20,21]. In this regime, the resistance of the material to shear and plastic deformation becomes the critical performance attribute. In contrast, at normal or near-normal incidence (~90°), the erosion mechanism shifts to being controlled by brittle fracture phenomena [22,23]. This includes the initiation and propagation of subsurface cracks, debonding at the fiber–matrix interface, and the chipping or fracture of the material’s constituents. In this impact-dominated regime, the critical performance-defining properties are the material’s fracture toughness and its ability to dissipate energy [24,25].

The different mechanisms involved lead to significantly different erosion responses in monolithic GFRP and CFRP systems. The higher hardness and stiffness of carbon fibers give CFRPs an advantage in conditions with low impact angles, where shear forces are dominant. However, CFRPs are also more brittle, which becomes a disadvantage in situations involving high-angle impacts. In contrast, glass fibers possess greater fracture toughness [26], allowing GFRPs to perform better under normal impact conditions by more effectively dissipating kinetic energy [27]. This advantage, however, is countered by their lower surface hardness, making them more vulnerable to micro-cutting mechanisms that occur at low impact angles. This inherent trade-off between hardness and toughness creates a fundamental design challenge that must be addressed when developing robust, erosion-resistant composite materials.

In an effort to overcome this challenge, significant research in the last decade has focused on the modification of the PMC matrix with nanoscale fillers [28]. Among these, Carbon Nanotubes (CNTs) have emerged as a leading candidate for matrix reinforcement [29,30,31,32,33,34], owing to their extraordinary mechanical properties and high aspect ratio.

While numerous studies have demonstrated that CNT incorporation can enhance the tribological performance of composites [35,36,37,38] through mechanisms such as increasing matrix hardness and hindering nanoscale crack propagation, its influence on solid particle erosion is not unequivocally beneficial [34]. In fact, the outcomes reported in the literature vary considerably, highlighting several countervailing phenomena. Agglomerates resulting from inadequate dispersion, for instance, can act as stress concentration sites that precipitate premature failure [37]. More critically, the embrittlement of the matrix—a well-documented consequence of restricted polymer chain mobility—can significantly reduce a composite’s overall impact toughness [33,39,40]. This degradation is particularly detrimental under the impact-dominated erosion conditions prevalent at high impingement angles [41].

A significant gap in knowledge exists in the literature at this stage. Most existing studies have primarily focused on the effects of nano-reinforcement within a single, monolithic composite system (such as laminates where all reinforcement layers are of the same type, e.g., pure E-glass fiber [S1]) [29,31,34,36,41,42,43,44]. Although we understand certain individual phenomena related to nano-reinforcement, there remains a significant knowledge gap concerning the complex and additive interactions within a multi-scale hybrid structure. Specifically, the fundamental principles that govern the interactions between the advantages and disadvantages of nano-reinforcement and the inherent macro-scale differences in a hybrid system—such as the stiff yet brittle layers of carbon fiber-reinforced polymer (CFRP) compared to the compliant yet tough layers of glass fiber-reinforced polymer (GFRP)—remain unclear. It is uncertain whether the addition of multi-walled carbon nanotubes (MWCNTs) will improve the erosion resistance of both materials, or if the nano-scale embrittlement will disrupt the delicate macro-scale synergy of the hybrid structure. This uncertainty presents a significant barrier to the rational design of advanced composites for applications where erosion resistance is critical.

The primary novelty of this research is not only in characterizing new combinations of materials but also in systematically examining the synergistic and antagonistic interactions that occur within these multi-scale systems. To our knowledge, this study provides the first comprehensive comparative analysis of the effects of MWCNT reinforcement on monolithic GFRP, monolithic CFRP, and their hybrid configurations in relation to erosive wear. This analysis employs a parametric experimental approach, where the impingement angle is systematically varied (30°, 45°, 60°, and 90°) to assess the material response across a spectrum from shear-dominated to impact-dominated wear regimes. Another objective is to clarify and identify the specific conditions under which nano-reinforcement is beneficial or detrimental. By addressing this complex performance dichotomy, the findings of this study aim to provide a more nuanced and mechanistically grounded framework for the tailored design of next-generation erosion-resistant composites.

## 2. Materials and Methods

### 2.1. Materials and Fabrication of Composites

**Figure 1:**
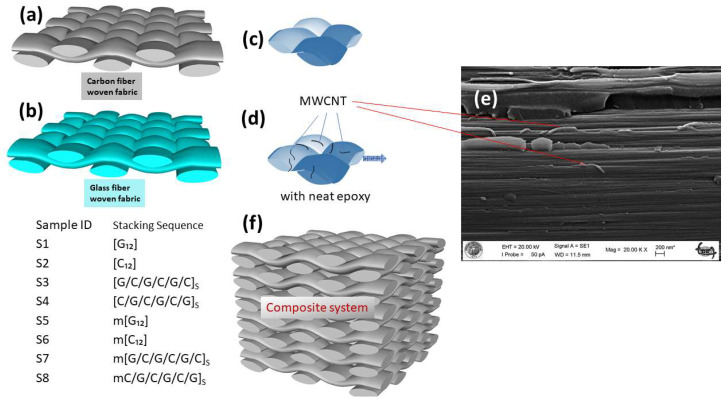
Schematic representation of the composite constituents and hierarchical structure: (**a**) Carbon fiber-woven fabric; (**b**) Glass fiber-woven fabric; (**c**) Fiber impregnation with neat epoxy matrix; (**d**) Fiber impregnation with MWCNTs-modified epoxy matrix; (**e**) SEM micrographs showing MWCNTs within composite; (**f**) Multi-layer composite laminate stack.

The materials used for fabrication are detailed in Table 1 and shown in Figure 1. The main reinforcements were commercial E-glass and high-strength carbon fiber fabrics (Dost Kimya, Istanbul, Turkiye), both with a plain-weave structure and a nominal areal weight of 200 g/m^2^ (Figure 1a,b). To ensure strong interfacial bonding, the materials were used in their as-received state, which included surface sizings applied by the manufacturer: an aminosilane agent for the E-glass and a standard epoxy-compatible coating for the carbon fabric. The polymer matrix was a two-component epoxy system based on Diglycidyl Ether of Bisphenol A (MGS L160/H260s) (Figure 1c). For the multi-scale composites, carboxyl-functionalized multi-walled carbon nanotubes (COOH-MWCNTs) (Timesnano, Chengdu, China) served as the nano-reinforcement. These COOH-MWCNTs, chosen for their excellent dispersion ability, had a purity of 95%, lengths between 10 and 30 µm, an outer diameter of 20–30 nm, and a specific surface area of 110 m^2^/g.

The fabrication protocol for all composite laminates commenced with the formulation of neat and reinforced matrix, wherein 0.3 wt.% of COOH-MWCNTs were dispersed into the epoxy resin via high-intensity soSnication conducted for 30 min within an ice bath to preclude thermal degradation (Figure 1d,e). Following the incorporation of a stoichiometric amount of hardener, the 12-ply preforms were constructed by progressive impregnation according to their prescribed stacking sequences (in Table 2) and then consolidated under a negative pressure of 0.8 bar in a vacuum bag assembly to minimize void content (Figure 1f). The cure cycle was subsequently executed under continuous vacuum, involving a precisely controlled two-stage thermal process: an initial one-hour dwell at 70 °C designed to reduce resin viscosity and facilitate complete fiber wet-out, followed by a four-hour post-cure at 110 °C to ensure the achievement of full matrix cross-linking and optimal thermomechanical properties.

To form the basis of this study, four distinct laminates, each comprising 12 plies, were produced. These laminates include two monolithic reference specimens: a pure carbon fiber laminate, designated as [C_12_], and a pure glass fiber laminate, [G_12_]. In addition, two symmetric, interlayer sandwich-type hybrid configurations were designed: one with glass fiber outer surfaces, [G/C/G/C/G/C]_s_, and another with carbon fiber outer surfaces, [C/G/C/G/C/G]_s_. The subscript ‘s’ denotes symmetry with respect to the laminate’s mid-plane. Crucially, each of these four configurations was fabricated in two distinct versions: a baseline version using neat epoxy and a second version reinforced with MWCNTs. Thus, eight different material types were produced, as shown in Table 2 with sample ID (identification) for comparative analysis.

**Figure 2:**
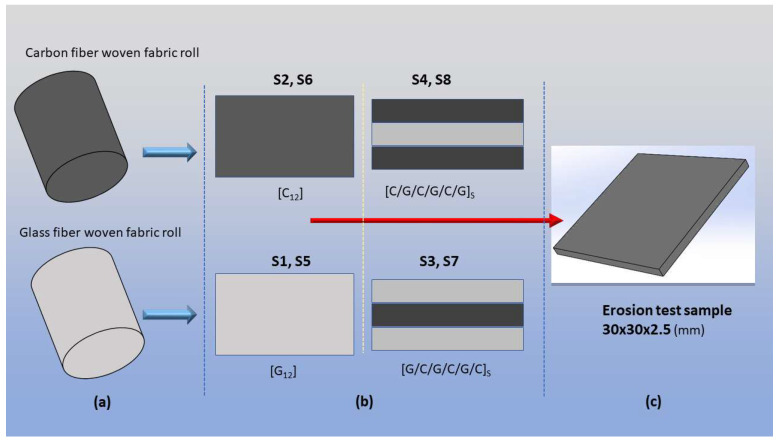
Test sample preparation: (**a**) fiber rolls; (**b**) composite laminate production; (**c**) test sample dimensions.

Individual fabric plies were first tailored from the as-received material rolls and systematically laid up to form the prescribed 12-ply laminates, as shown in Figure 2a,b. Upon completion of the vacuum-assisted curing cycle, these fully cured plates were sectioned into the final specimen geometries (as seen in Figure 2c) using a diamond-grit circular saw.

### 2.2. Mechanical Tests

#### 2.2.1. Tensile Test

The quasi-static tensile properties of the laminates were characterized pursuant to ASTM D3039 [45] standard. The specimens with nominal dimensions of 250 × 25 × 2.5 mm were tested on an Instron 8801 universal testing machine under displacement control at a constant crosshead speed of 2 mm/min. To ensure reliability, all reported tensile properties represent the average of three replicate tests for each composite configuration.

#### 2.2.2. Hardness Measurement and Density Measurement

The characterization of the laminates involved quantification of both hardness and density, following their respective standard testing methods. Hardness was measured using a Barcol impressor, in strict adherence to the ASTM D2583-25 [46] protocol. To ensure a statistically reliable representation of the surface properties, the reported hardness for each laminate is the arithmetic mean of at least ten measurements. Concurrently, the density of the composites was determined using the immersion method, as outlined in ASTM D792-20 [47].

### 2.3. SPE Test

**Figure 3:**
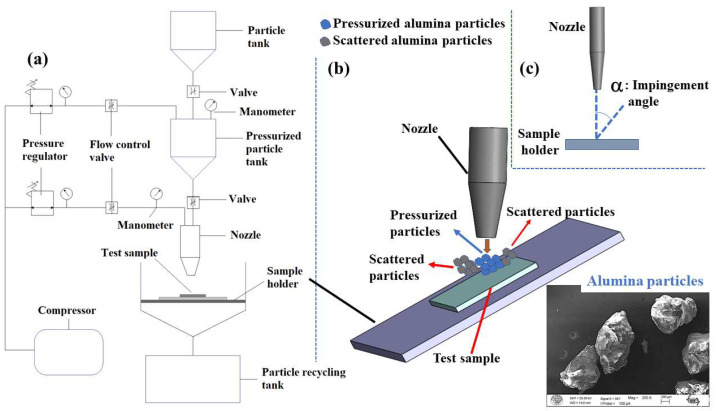
Erosion test configuration: (**a**) Schematic test setup; (**b**) Particle and sample representation during erosion testing; (**c**) Impingement angle definition.

The erosive wear response of the composite laminates was assessed using a custom-built testing apparatus designed in accordance with the ASTM G76-18 [48] standard, as shown in Figure 3a. The system enables the controlled impingement of a particle flux onto a test specimen by incorporating three critical subsystems: a particle feeding mechanism, an acceleration nozzle, and a specimen positioning unit.

The erosive wear evaluation was conducted using angular alumina (Al_2_O_3_) particles as the erodent, the properties and morphology of which are detailed in Table 3 and Figure 3b, respectively. This material, with a nominal size of ~600 µm, was specifically selected for its high hardness and angular geometry, attributes known to elicit pronounced and repeatable wear phenomena. As depicted schematically in Figure 3a, the experimental protocol involved accelerating a 2 kg charge of these erodent particles onto a stationary specimen (Figure 3b) via a pressurized air stream.

In line with the research objectives, while the particle impact velocity was consistently maintained at a constant value of 34 m/s through precise pressure regulation for all tests, the impingement angle was deliberately varied to analyze the sensitivity of the erosion mechanism. Specifically, the orientation between the particle stream and the specimen surface was set to 30°, 45°, 60°, and 90°, with this angular positioning accomplished using the specialized holder configurations depicted in Figure 3c. To ensure the reliability of the SPE test results, three replicate tests were conducted for each sample configuration.

To assess the resulting erosive damage, the mass of each specimen was measured both before and after testing using a precision balance with a sensitivity of 10^−4^ g. The erosion performance of the material was evaluated based on the Erosion ER. The primary indicator of performance, the ER, is defined in Equation (1) as the ratio of the total mass loss of the test specimen (ΔW) to the total mass of the impinging abrasive particles (Qp). The results are presented as a dimensionless ER in g/g × 10^−7^, which allows for a comparative interpretation of the material’s wear resistance. The dimensions of the erosion test sample are 30 × 30 × 2.5 (mm) as shown in Figure 2c.

(1) $$ER=\frac{\Delta W}{Qp}$$

## 3. Results and Discussion

### 3.1. Mechanical Test Results

#### 3.1.1. Tensile Test Results

**Figure 4:**
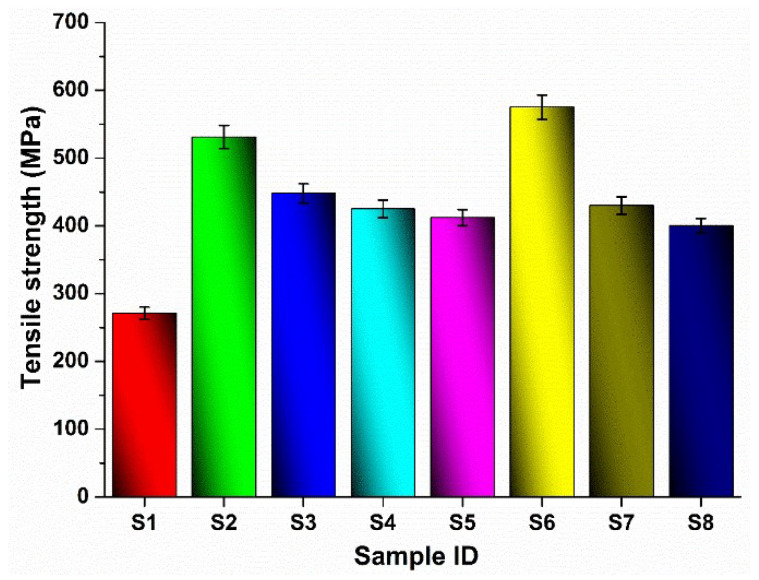
Tensile test results.

The laminate architecture dictates the influence of MWCNT reinforcement on tensile strength (UTS). While the nano-reinforcement provides a notable strengthening effect in the monolithic GFRP and CFRP systems (S1, S2, S5, S6) [49,50,51], a starkly contrasting, detrimental outcome is observed in all hybrid configurations (S3, S4, S7, S8) as seen in Figure 4, consistent with the literature [52,53,54]. This performance reversal is attributed to the inherent strain incompatibility that governs the tensile response of the hybrid systems: the high-stiffness, low-elongation carbon fibers fracture prematurely, precipitating a failure of the entire laminate long before the more compliant glass fibers can be fully loaded [53]. Consequently, any potential matrix-strengthening benefit imparted by the MWCNTs is rendered ineffective, overshadowed by this dominant, architecture-dominated failure mode.

#### 3.1.2. Hardness and Density Test Results

An analysis of the Barcol hardness data in Table 4 highlights the significant reinforcing effect of MWCNTs incorporation. The addition of MWCNTs to the epoxy resin increased the matrix hardness by approximately 60%. This improvement at the matrix level led to enhanced mechanical properties of the composite laminates, which align with findings in existing literature [55,56,57].

A comparison between the unreinforced GFRP (S1) and its MWCNT-modified counterpart (S5) reveals a modest 2.5% increase in hardness, but more critically, a significant 52% augmentation in its ultimate tensile strength (UTS). Similarly, the reinforcement of the CFRP system (S6 vs. S2) yielded increases of 1.5% in hardness and 8.5% in UTS, respectively. Crucially, this demonstrated improvement in mechanical integrity directly correlated with enhanced tribological performance, manifesting as a consistent and quantifiable reduction in the ER for the MWCNTs-reinforced specimens. The trend of slight hardness improvements persisted in the sandwich-type hybrid systems.

### 3.2. SPE Test Results

**Figure 5:**
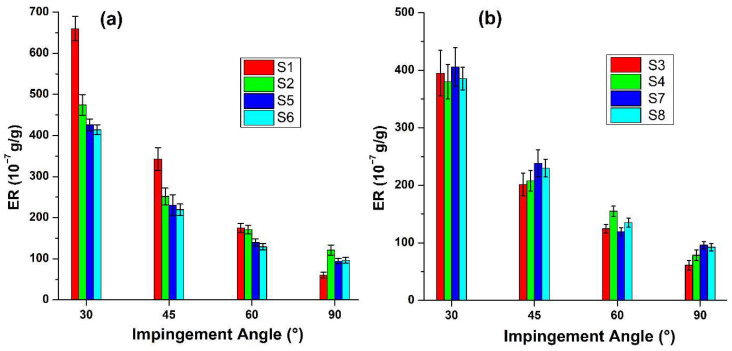
SPE test results: (**a**) Results of samples S1, S2, S5, and S6; (**b**) Results of samples S3, S4, S7, and S8.

An analysis of the erosion behavior for the monolithic composite laminates (S1, S2, S5, and S6) shows a classic ductile response. This behavior is characterized by a peak ER occurring at a low impingement angle of 30°, while the minimum ER is observed at an impingement angle of 90° (Figure 5a,b).

Consistent with the established literature [58,59] for ductile polymer composites, the peak ER was recorded for the GFRP specimens (S1) at the 30° impingement angle. This is a direct consequence of a wear mechanism governed by the predominance of the tangential particle velocity component. While the normal velocity component induces localized indentation and sub-surface stress, it is this tangential, shear-dominated action that dictates the material removal process through micro-cutting and plowing. As illustrated by the resultant surface topographies of specimens S1 and S2, this leads to the efficient excavation of the matrix, the fracturing and subsequent dislodgement of near-surface fibers, and ultimately, the trenching and severing of deeper-lying reinforcement, thereby creating extensive and directionally aligned damage grooves.

The impact of MWCNT reinforcement in the glass fiber composite (designated as S5) is more complex and varies with the impingement angle. The MWCNTs improved erosion resistance at low to moderate angles (ranging from 30° to 60°) compared to the GFRP, designated as S1. However, this trend changed significantly at normal incidence (90°). In this case, the MWCNT-modified GFRP (S5) showed a higher ER, indicating a reduction in performance under conditions dominated by impact.

The data presented for the intermediate 45° and 60° angles provides further crucial insight into the wear behavior across the shear-to-impact dominated transition. For all specimens, a decrease in the ER is observed as the impingement angle increases from 30° toward 90°. In the monolithic systems (Figure 5a), this trend is particularly pronounced for the tougher S1 (GFRP) specimen, which relies heavily on its ability to dissipate shear energy. The harder S2 (CFRP) specimen, while exhibiting lower overall ER at low angles, shows a comparatively flatter downward trend, indicating its greater inherent resistance to cutting is less sensitive to small changes in angle. The MWCNT-reinforced specimens (S5 and S6) and sandwich-type hybrid specimens follow similar trajectories to S1 and S2 but with a generally lower magnitude of wear, reinforcing the conclusion that the nano-filler primarily enhances resistance to the erosive wear mechanisms that are still significantly active at these intermediate angles.

This predictable decrease in ER is entirely consistent with a progressive, mixed-mode wear mechanism. As the angle of incidence increases, the tangential velocity component of the particles diminishes, thereby reducing the efficacy of micro-cutting and ploughing. Simultaneously, the normal velocity component increases, escalating the contribution of subsurface impact damage and strain hardening to the overall material loss. The smooth and logical nature of this transition, evident across both monolithic and sandwich-type hybrid systems (Figure 5a,b), expresses the importance of concentrating the detailed morphological analysis on the 30° and 90° angles, as these represent the well-defined mechanistic endpoints of this behavioral spectrum.

This deleterious effect at 90° is attributed to the inherent embrittlement of the polymer matrix following the addition of MWCNTs; with this situation, hardness also increases with reinforcement. This increased brittleness renders the matrix more susceptible to crack initiation and propagation when subjected to the high-energy, normal-impact forces characteristic of 90° impingement, thereby accelerating material loss.

Similarly, to their monolithic counterparts, the sandwich-type hybrid composites (S3, S4) demonstrated a ductile failure mode, with erosion rates peaking at a 30° angle of incidence (Figure 5b).

The data in Figure 5b reveals a critical finding: at normal incidence (90°), the simultaneous incorporation of carbon fiber and MWCNTs into the glass-epoxy system (specimens S7 and S8) precipitates a significant increase in the Erosion Rate. This behavior strikingly mirrors the performance degradation previously observed in the MWCNT-modified GFRP system (S5) at the same impact angle.

This observed increase in material loss can be attributed to a compounding embrittlement effect. The combination of inherently brittle carbon fibers with the matrix embrittlement induced by the MWCNTs creates a hybrid system with substantially reduced fracture toughness. Such systems are acutely vulnerable to the impact-dominated, brittle failure mechanisms that govern material removal at normal incidence, where the capacity to absorb and dissipate kinetic energy is paramount. Consequently, the composite structure, with its enhanced stiffness but diminished toughness, loses the ability to effectively arrest the subsurface cracking initiated by each impact, leading to accelerated material loss.

By integrating the performance results across all tested angles, we can clearly establish a hierarchy of erosion resistance. The neat hybrid composites (S3 and S4) showcased the most robust and balanced overall performance with high erosion resistance. Each exhibited specific advantages: S4 excelled in shear-dominated environments, while S3 performed better in impact-dominated scenarios.

In stark contrast, the monolithic S1 (GFRP) specimen highlighted the performance differences dictated by wear mechanisms. It exhibited a significantly higher ER under the critical shear-dominated 30° condition, primarily due to its lower surface hardness and resistance to cutting. Conversely, at the 90° impact angle, S1 demonstrated one of the lowest and most favorable ER. This favorable performance resulted from its superior fracture toughness and ability to dissipate impact energy.

This observation emphasizes that while a singular material property can be highly beneficial in a specific context, a well-designed hybrid structure can offer superior and more versatile performance across a broader range of erosive conditions.

### 3.3. Damage Mechanisms

**Figure 6:**
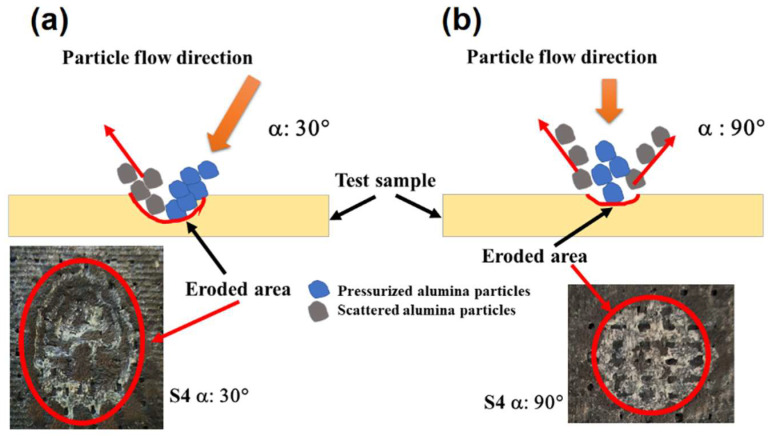
Eroded area: (**a**) low impingement angle; (**b**) high impingement angle.

Figure 6a,b provide a schematic representation comparing the key damage mechanisms at impingement angles of 30° and 90°. At the lower angle of 30°, the interaction between particles is primarily influenced by shear forces [60], leading to a surface topography marked by broad, laterally excavated damage zones (see Figure 6a).

**Figure 7:**
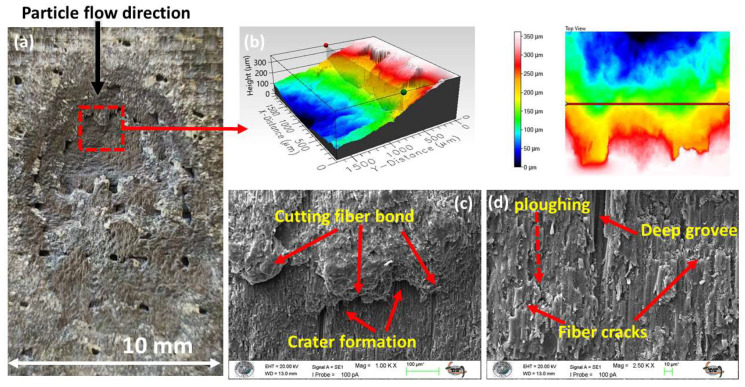
Damage formation type of S3 sample at 30° impingement angle: (**a**) macro image of eroded area; (**b**) 3D optical profile image of the eroded surface in the deepest region; (**c**) 1.00 KX zoom at the deepest region; (**d**) 2.50 KX zoom at the deepest region.

The topographical result of this ductile erosion regime is evident in the S3 sandwich-type hybrid specimen, where extensive, deep cavities form on the eroded surface (Figure 7a). A high-magnification view of the surface topography within one such cavity, acquired via 3D optical profilometry, is presented in Figure 7b.

This complex morphology is a direct consequence of a wear mechanism dictated by the high tangential velocity of the erodent particles [61,62]. This kinetic component drives two simultaneous damage modes at the surface: micro-cutting, which fractures the reinforcement and creates micro-cantilever structures (Figure 7c), and ploughing, which excavates and displaces the matrix material (Figure 7d). The initial effect of these mechanisms is the efficient removal of the protective polymer matrix, leading to the direct exposure of the underlying reinforcement fibers [63].

Deprived of their foundational support by the removal of the surrounding matrix, the exposed fibers become highly susceptible to fracture and dislodgement by the shear and bending forces imparted by succeeding particle impacts. This sequential failure process—entailing matrix excavation followed by fiber fracture—constitutes the primary mechanism of material loss under the ductile regime and comprehensively explains the high ER observed at low impingement angles.

**Figure 8:**
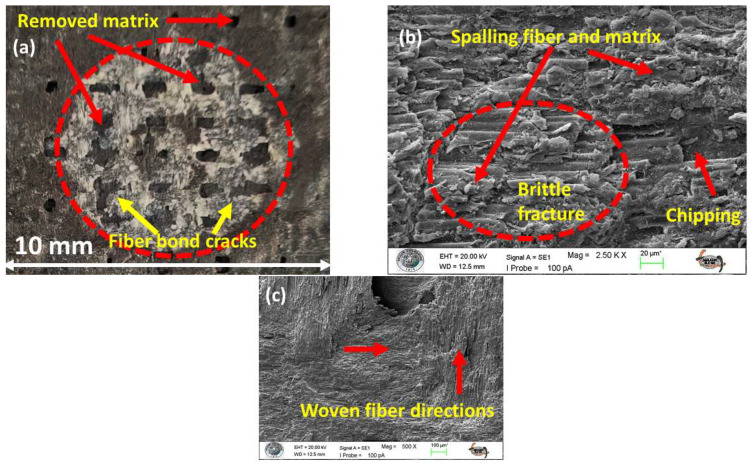
Damage formation type of S4 sample at 90° impingement angle: (**a**) macro image of eroded area; (**b**) 1.00 KX zoom; (**c**) 500 X zoom.

When an impact occurs at a 90° angle, it results in a unique damage pattern typical of brittle failure. This process often initiates with the formation of cone cracks that form beneath the surface (see Figure 8a). The eventual detachment of these cracks leads to the noticeable material removal process known as chipping and spalling (Figure 8b,c) [64].

Microstructural examination reveals a complex network of matrix cracks, typically including radial cracks emanating from the impact center and circumferential cracks forming around it (Figure 8a). These fractures propagate to the fiber–matrix boundary, causing extensive interfacial debonding, leaving unsupported fibers exposed in clean troughs (Figure 8a,c). With their lateral support eliminated, these fibers become highly susceptible to direct brittle fracture. In high-energy impacts, this can manifest as trans-laminar cracks that cut directly through the fiber and matrix layers.

### 3.4. Effect of Hybridization of Composites on SPE

A comparative analysis of the SPE performance, summarized in Table 5 and Table 6, reveals a clear performance trade-off that is primarily determined by the impingement angle and the selected material benchmark. When compared to GFRP (S1), which demonstrates superior toughness, the advantages of hybridization are limited to the shear-dominated 30° angle. At this angle, the incorporation of harder carbon fibers consistently reduces the ER significantly, with improvements of up to 42% observed (S4, S8). However, this benefit is completely reversed at normal incidence (90°), where the brittleness of both the carbon fibers and the MWCNTs-modified matrix becomes detrimental. The sandwich-type hybrid systems (S7, S8) exhibit a severe performance degradation, with the ER increasing by as much as 60% compared to the GFRP (S1).

Conversely, using the brittle CFRP (S2) as the reference point showcases the effectiveness of hybridization as a toughening strategy. While low-angle erosion is moderately reduced (up to 20% for S4), the most significant enhancement is realized at 90°. In this case, the strategic interleaving of glass fibers (S3) serves as a critical mechanism for damage mitigation, resulting in an impressive ~50% reduction in material loss. This analysis also confirms the dual nature of the MWCNTs; while still offering an improvement over pure CFRP, their embrittling effect attenuates the profound synergistic benefit observed in the hybrid configurations.

### 3.5. Effect of Hardness and Tensile Strength of Composites on SPE.

The Pearson correlation coefficient (*R*) quantifies the linear relationship between two variables, ranging from −1 (a perfect negative association) to +1 (a perfect positive association), with 0 indicating the complete absence of a linear relationship. The magnitude of the coefficient, |r|, determines the strength of the association, which is typically categorized as weak (|r| < 0.3), moderate (0.3 ≤ |r| < 0.7), and strong (|r| ≥ 0.7) [65]. The analysis reveals a strong positive correlation between hardness and tensile strength, confirming that these properties increase in tandem. As the preceding experimental results have shown that an enhancement in either property improves erosion resistance, a corresponding negative correlation for the Hardness-ER and UTS-ER data pairs is both expected and statistically validated by the analysis in Table 7. Notably, the magnitude of the coefficient is greatest for the Hardness-ER pairing, suggesting that surface hardness (with *R* = −0.758422) is the most influential property governing the ductile erosion response of these composites.

It is important to note that while these property correlations are strong, the overall erosion performance is significantly influenced by structural factors, specifically the effects of hybridization and the reinforcement of MWCNTs.

(2) $$R(X,\;Y)=\frac{\sum (x-\bar{x})\;(y-\bar{y})}{\sqrt{\sum {\left(x-\bar{x}\right)}^{2}\Sigma {\left(y-\bar{y}\right)}^{2}}}$$

### 3.6. Morphology of Eroded Surfaces

**Figure 9:**
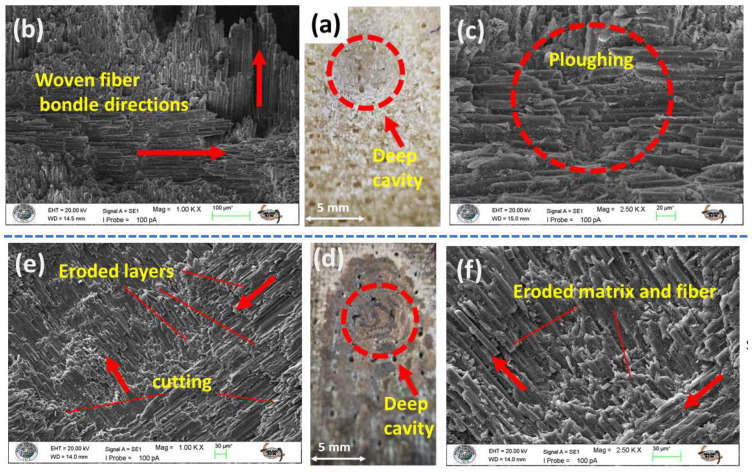
Eroded surface analysis of S1–S2 test samples at 30° impingement angle: (**a**) macro image of S1 sample eroded area; (**b**) 1.00 KX zoom of S1 sample eroded area; (**c**) 2.50 KX zoom of S1 sample eroded area; (**d**) macro image of S2 sample eroded area; (**e**) 1.00 KX zoom of S2 sample eroded area; (**f**) 2.50 KX zoom of S2 sample eroded area.

**Figure 10:**
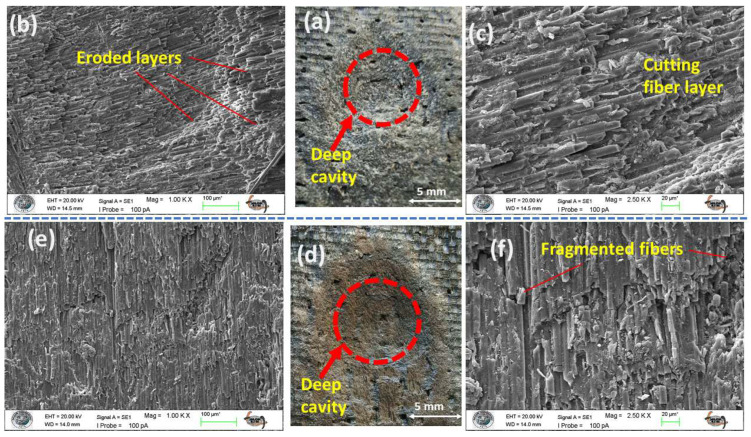
Eroded surface analysis of S5–S6 test samples at 30° impingement angle: (**a**) macro image of S5 sample eroded area; (**b**) 1.00 KX zoom of S5 sample eroded area; (**c**) 2.50 KX zoom of S5 sample eroded area; (**d**) macro image of S6 sample eroded area; (**e**) 1.00 KX zoom of S6 sample eroded area; (**f**) 2.50 KX zoom of S6 sample eroded area.

The wear morphologies resulting from the low 30° impingement angle (Figure 9a,d and Figure 10a,d), where erosion is governed by shear-dominated mechanisms such as micro-cutting and ploughing, illustrated in Figure 9b,c and Figure 10e,f. Macro and scanning electron microscope (SEM) analysis of the monolithic specimens (S1, S2) and their MWCNT-reinforced counterparts (S5, S6) provides compelling visual evidence of this ductile wear regime. A key characteristic of this wear process is the oblique cutting of the fiber bond, which aligns with the trajectory of the impacting particles, as clearly shown in Figure 9e and Figure 10b,c. The combined action of micro-cutting and micro-ploughing results in significant excavation of the matrix material (depicted in Figure 9a,d and Figure 10a,d), followed by the fracturing of the angles (shown in Figure 10f) and dislodgement of the reinforcement. This sequential process leads to the formation of deep, elongated cavities or troughs, which are prominent topographical features in the wear scars (as seen in Figure 9a,d and Figure 10a,d). Additionally, comminuted debris, consisting of fragmented fibers and pulverized matrix material, is evident across the worn surfaces (illustrated in Figure 9f and Figure 10f).

A notable distinction emerges, however, upon comparing the MWCNT-reinforced laminates (S6) with their neat counterparts (S2). The nano-reinforcement appears to promote a more uniform wear, yielding a markedly smoother wear surface at the micro-scale (Figure 9e and Figure 10e). The macroscopic consequence of the enhanced erosion resistance imparted by the MWCNTs in both the S5 and S6 samples is manifest as a clear reduction in the overall severity and depth of the damage.

**Figure 11:**
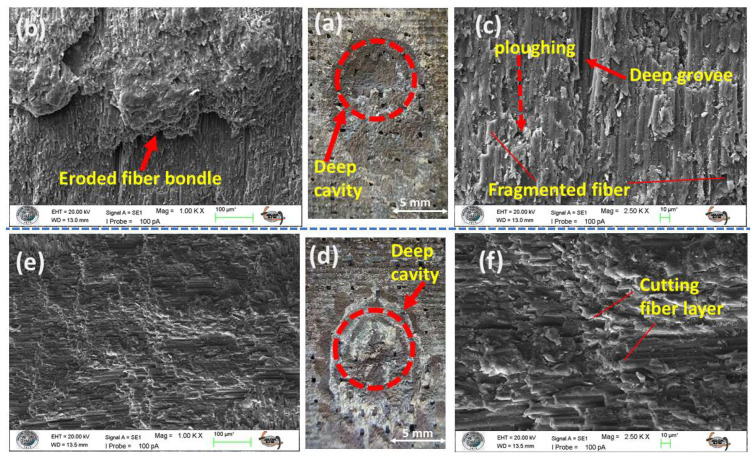
Eroded surface analysis of S3–S4 test samples at 30° impingement angle: (**a**) macro image of S3 sample eroded area; (**b**) 1.00 KX zoom of S3 sample eroded area; (**c**) 2.50 KX zoom of S3 sample eroded area; (**d**) macro image of S4 sample eroded area; (**e**) 1.00 KX zoom of S4 sample eroded area; (**f**) 2.50 KX zoom of S4 sample eroded area.

**Figure 12:**
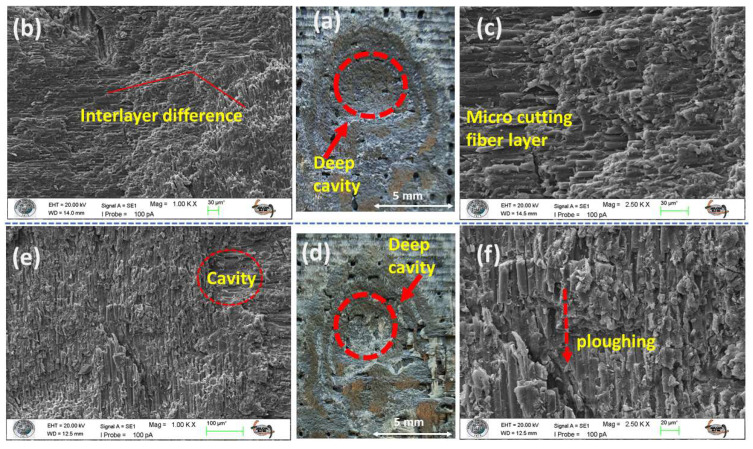
Eroded surface analysis of S7–S8 test samples at 30° impingement angle: (**a**) macro image of S7 sample eroded area; (**b**) 1.00 KX zoom of S7 sample eroded area; (**c**) 2.50 KX zoom of S7 sample eroded area; (**d**) macro image of S8 sample eroded area; (**e**) 1.00 KX zoom of S8 sample eroded area; (**f**) 2.50 KX zoom of S8 sample eroded area.

The wear morphologies of the hybrid composites under the ductile erosion regime are illustrated in Figure 11 and Figure 12. In line with the previously observed shear-dominated failure, the main feature of damage is the formation of deep cavities (see Figure 11a,d and Figure 12a,d). These cavities result from a combination of micro-cutting and plowing actions that excavate both the matrix and the reinforcement (as shown in Figure 11b,c,e,f and Figure 12b,c,e,f). Upon macroscopic examination of the wear scars in the hybrid systems, a distinct layered morphology is evident, where the boundaries between the alternating glass and carbon fiber laminae are clearly visible (Figure 11d and Figure 12d). This observation visually confirms that erosive wear progresses layer by layer through the structure. On a microscopic level, the damage patterns observed across all four sandwich-type hybrid configurations (S3, S4, S7, S8) demonstrate remarkable similarity, which corresponds directly with their closely clustered erosion resistance (ER) values at low impingement angles.

The consistent performance observed is primarily due to the overwhelming influence of particle kinetic energy, which is mainly transferred as shear and plastic deformation. This shear-dominated action rapidly erodes the matrix, exposing the underlying fiber tows and particularly the vulnerable interlaced nodes of the fabric weave (Figure 11b). As the particle flux continues, it compromises the fiber–matrix interfacial bond, progressively weakening both the reinforcement and the matrix, ultimately leading to the dislodgement of both components from the system. Importantly, under these shear-dominated conditions, a positive synergistic effect arises across all hybrid systems, resulting in an erosion resistance that surpasses that of any monolithic benchmark.

**Figure 13:**
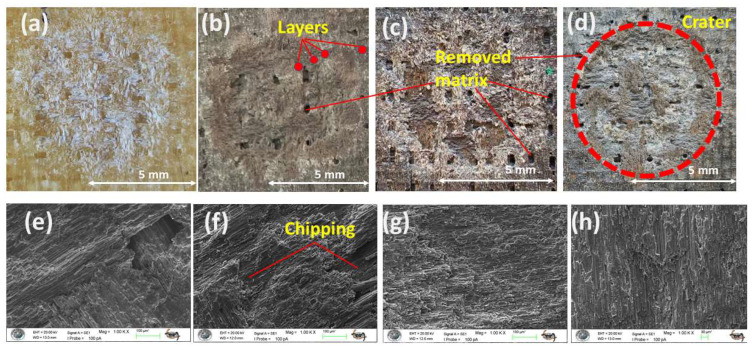
Macro images of eroded surfaces of test samples at 90° impingement angle: (**a**) S1; (**b**) S2; (**c**) S7; (**d**) S8; (**e**) 1.00 KX zoom of S1; (**f**) 1.00 KX zoom of S2; (**g**) 1.00 KX zoom of S7; (**h**) 1.00 KX zoom of S8.

Increasing the impingement angle to 90° fundamentally changes the erosive wear mechanism by reducing the tangential velocity component and maximizing the normal force of the particle impacts. As a result, the shear-dominated wear, which is typical of low-angle erosion, is minimized. Instead, the material removal process is governed by compressive stresses and repeated impact loading, leading to localized plastic deformation, strain hardening, and ultimately, brittle fracture (as illustrated in Figure 13a–d). This transition results in a wear scar, defined not by elongated grooves but by the formation of localized craters and surface chipping, as clearly shown in Figure 13b.

The disparate responses of the monolithic laminates to this impact-dominated regime underscore their fundamental mechanical differences. The neat GFRP (S1), owing to its superior toughness and inherent capacity for energy dissipation, effectively mitigates impact damage, resulting in minimal material loss (Figure 13a). In stark contrast, the intrinsically brittle nature of the pure CFRP (S2) renders it highly vulnerable to normal impacts. Its limited ability to absorb energy precipitates extensive damage via chipping and spalling mechanisms, leading to substantially higher ER (Figure 13b).

In the sandwich-type hybrid composites (S7, S8), a synergistic behavior is observed under the specified conditions. Their erosion performance falls between that of the two monolithic benchmarks (see Figure 13c,d). The tougher glass fiber layers help to halt catastrophic crack propagation, which would typically characterize the response of CFRP. Meanwhile, the stiffer carbon fibers provide structural support. This combination results in a balanced and more damage-tolerant sandwich-type hybrid structure. Regardless of their specific composition, all specimens that experienced brittle erosion at normal incidence exhibited a characteristic surface morphology marked by chipping and spalling (as illustrated in Figure 13e–h).

## 4. Conclusions

This investigation provides a comprehensive analysis of the SPE response of monolithic, sandwich-type hybrid, and multi-scale composites, revealing a profound performance dichotomy dictated by the governing wear mechanism. The study confirms that a material’s architectural design and its constituent properties must be synergistically aligned with the anticipated service environment, as design choices beneficial for one wear regime can be severely detrimental in another.

The central and novel contribution of this work is its quantitative analysis of the dual role of multi-scale reinforcement, which is dictated by the dominant wear mechanism. Specifically, in the shear-dominated regime characterized by a 30° impingement angle, the data clearly demonstrates that increasing material hardness and resistance to micro-cutting—achieved through either the addition of MWCNTs or the incorporation of stiffer carbon fibers—results in a consistent and predictable reduction in erosion, by up to 42%.

The observed progressive, mixed-mode transition at the intermediate impingement angles of 45° and 60° for all configurations further supports the robustness of this shear-dominated model. It shows a steady decrease in erosion as the mechanisms gravitate toward an impact-dominated response.

However, the most critical findings emerge in the 90° impact-dominated regime, revealing significant implications for existing literature. While strategically hybridizing the brittle CFRP led to an impressive reduction in wear by approximately 50%, this study provides comparative evidence that this beneficial synergy can be negated or even reversed through nano-scale matrix modification. The data indicate that reinforcing tough monolithic GFRP with MWCNTs resulted in a catastrophic increase of about 60% in impact-induced erosion.

This outcome highlights a fundamental principle for multi-scale composite design: in the context of impact erosion, the macroscopic toughness necessary for energy dissipation is crucial. Any nano-scale enhancement that compromises this toughness—regardless of improvements in static strength or hardness—can ultimately lead to a more vulnerable material system.

It is essential to interpret the findings herein within the context of the study’s specific limitations. The established material performance hierarchies are intrinsically linked to the experimental conditions, namely, a singular erodent type (angular alumina), room temperature, and a constant impact velocity. Consequently, the observed dominance of either shear or impact-dominant wear mechanisms may shift significantly under different kinetic energy regimes or with erodents of varying geomechanical properties, potentially altering the performance rankings. Finally, the negative synergy observed in the multi-scale hybrids is specific to this material combination; it is conceivable that a different matrix formulation or nano-filler concentration could mitigate this effect and yield a more favorable outcome, warranting further investigation in subsequent studies.

## Figures and Tables

**Table 1:** Properties of composite constituent materials (from manufacturer data sheets).

Material	Tensile Strength (MPa)	Modulus of Elasticity (GPa)	Density (g/cm^3^)	Fiber Diameter
**Epoxy resin**	70	3.2	1.19	-
**Glass fiber fabric**	2600	72	2.5	9 (μm)
**Carbon fiber fabric**	3800	238	1.8	7 (μm)

**Table 2:** Nomenclature and stacking sequences of the fabricated laminates.

Sample ID	Stacking Sequence	Description	Matrix Type
S1	[G_12_]	Pure Glass Laminate	Neat Epoxy
S2	[C_12_]	Pure Carbon Laminate	Neat Epoxy
S3	[G/C/G/C/G/C]_s_	Symmetric Hybrid (Glass-outer)	Neat Epoxy
S4	[C/G/C/G/C/G]_s_	Symmetric Hybrid (Carbon-outer)	Neat Epoxy
S5	[G_12_]	Multi-scale Glass Laminate	0.3% MWCNT-Epoxy
S6	[C_12_]	Multi-scale Carbon Laminate	0.3% MWCNT-Epoxy
S7	[G/C/G/C/G/C]_s_	Multi-scale Hybrid (Glass-outer)	0.3% MWCNT-Epoxy
S8	[C/G/C/G/C/G]_s_	Multi-scale Hybrid (Carbon-outer)	0.3% MWCNT-Epoxy

**Table 3:** Erosion test parameters.

Test Parameters	Description
**Erodent**	Aluminum Oxide (Al_2_O_3_)
**Erodent size**	~600 μm
**Erodent shape**	Angular
**Hardness of erodent**	Mohs 9
**Impingement angles**	30°/45°/60°/90°
**Impact velocity**	34 m/s
**Test temperature**	~25 °C
**Nozzle to sample distance**	10 mm
**Nozzle diameter**	6 mm
**Total abrasive weight**	2 kg

**Table 4:** Barcol hardness and density test results of composites.

Sample ID	Barcol Hardness (Average)	Standard Deviation of Hardness	Density (g/cm^3^)
Neat Epoxy	25.25	1.26	1.19
Neat Epoxy + MWCNT	38.14	6.23	1.2
S1	74.08	11.46	1.49
S2	81.58	3.92	1.31
S3	81	6.48	1.37
S4	82	6.37	1.35
S5	75.83	4.65	1.49
S6	82.67	5.99	1.35
S7	81.67	5.31	1.36
S8	82.5	4.03	1.36

**Table 5:** Test results of composites.

Impingement Angle			ER Results of Samples (g/g × 10^−7^)					
	S1	S2	S3	S4	S5	S6	S7	S8
**30°**	660	474.5	395	380	426	414	406	385.5
**90°**	60	121.3	61	78.5	94	96.5	96	92.5

**Table 6:** Comparative results on the effect of hybridization of composites on SPE.

Impingement Angle	The Percentage Change in the ER Results of Samples											
	S1–S3	S2–S3	S1–S4	S2–S4	S1–S7	S2–S7	S1–S8	S2–S8	S3–S7	S4–S7	S3–S8	S4–S8
**30°**	40%	17%	42%	20%	38%	14%	42%	19%	−3%	−7%	2%	−1%
**90°**	−2%	50%	−31%	35%	−60%	21%	−57%	24%	−57%	−22%	−52%	−18%

**Table 7:** Correlative results on the effect of hardness and tensile strength of composites on SPE.

Sample ID	Barcol Hardness	UTS	ER (at the 30°)	*R* (Correlation)		
**S1**	74.08	271	660	*Hardness-UTS*	*Hardness-ER*	*UTS-ER*
**S2**	81.58	531	474.5	0.71065197	−0.7584221	−0.5773
**S3**	81	448	395			
**S4**	82	425	380			
**S5**	75.83	412	426			
**S6**	82.67	575	414			
**S7**	81.67	430	406			
**S8**	82.5	400	385.5			

## Data Availability

The original contributions presented in this study are included in the article. Further inquiries can be directed to the corresponding author.

## Abbreviations

The following abbreviations are used in this manuscript:

MWCNT	Multi-walled carbon nanotube
SPE	Solid particle erosion
ER	Erosion rate
UTS	Ultimate tensile strength
SEM	Scanning electron microscope
CFRP	Carbon fiber-reinforced polymer
GFRP	Glass fiber-reinforced polymer
